# Identifying emerging trends in antimicrobial resistance using *Salmonella* surveillance data in poultry in Spain

**DOI:** 10.1111/tbed.13346

**Published:** 2019-09-13

**Authors:** Julio Alvarez, Gema Lopez, Petra Muellner, Cristina de Frutos, Christina Ahlstrom, Tania Serrano, Miguel A. Moreno, Manuel Duran, Jose Luis Saez, Lucas Dominguez, Maria Ugarte‐Ruiz

**Affiliations:** ^1^ VISAVET Health Surveillance Center Universidad Complutense Madrid Spain; ^2^ Departamento de Sanidad Animal Facultad de Veterinaria Universidad Complutense Madrid Spain; ^3^ Subdirección General de Sanidad e Higiene Animal y Trazabilidad Dirección General de la Producción Agraria Ministerio de Agricultura Pesca y Alimentación Madrid Spain; ^4^ Epi‐Interactive Wellington New Zealand; ^5^ Department of Veterinary Population Medicine College of Veterinary Medicine University of Minnesota St Paul USA; ^6^ Laboratorio Central de Veterinaria (LCV Algete) Ministerio de Agricultura Pesca y Alimentación Madrid Spain; ^7^ TRAGSATEC Tecnologías y Servicios Agrarios S.A Madrid Spain

**Keywords:** *antimicrobial resistance*, *foodborne*, *monitoring*, *Non‐typhoidal Salmonella*, *poultry*, *serotype Kentucky*

## Abstract

Despite of controls and preventive measures implemented along the food chain, infection with non‐typhoidal *Salmonella* (NTS) remains one of the major causes of foodborne disease worldwide. Poultry is considered one of the major sources of NTS. This has led to the implementation of monitoring and control programmes in many countries (including Spain) to ensure that in poultry flocks infection is kept to a minimum and to allow the identification and monitoring of circulating NTS strains and their antimicrobial resistance (AMR) phenotypes. Here, we investigated the information from the monitoring programme for AMR in *Salmonella* from poultry in Spain in 2011–2017 to assess the diversity in phenotypic resistance and to evaluate the programme's ability to detect multi‐resistance patterns and emerging strains in the animal reservoir. Data on serotype and AMR to nine antimicrobials obtained from 3,047 NTS isolates from laying hens (*n* = 1,060), broiler (*n* = 765) and turkey (*n* = 1,222) recovered during controls performed by the official veterinary services and food business operators were analysed using univariate and multivariate methods in order to describe host and serotype‐specific profiles. Diversity and prevalence of phenotypic resistance to all but one of the antimicrobials (colistin) were higher in NTS from broiler and turkey compared with laying hen isolates. Certain combinations of serotype and AMR pattern (resistotype) were particularly linked with certain hosts (e.g. susceptible Enteritidis with laying hens, multi‐drug resistant (MDR) Derby in turkey, MDR Kentucky in turkey and broiler). The widespread presence of certain serotype‐resistotype combinations in certain hosts/years suggested the possible expansion of MDR strains in the animal reservoir. This study demonstrates the usefulness of the analysis of data from monitoring programmes at the isolate level to detect emerging threats and suggests aspects that should be subjected to further research to identify the forces driving the expansion/dominance of certain strains in the food chain.

## 1 INTRODUCTION

Despite the control measures implemented in food production systems, foodborne infections remain one of the top causes of disease worldwide (Pires et al., 2015). Salmonellosis due to non‐Typhoidal *Salmonella* (NTS) is one of the leading causes of foodborne illness, with a global estimate of 129 million cases every year, of which 100,000 to 1 million result in death (mostly in immunocompromised patients) (Kirk et al., 2015). Even though incidence may be severely underestimated due to underreporting, levels of salmonellosis have lately remained stable in the United States (since the late 90’s) (Henao et al., 2015) and the European Union (since 2013) (EFSA & ECDC, 2018), thus highlighting the challenge posed by NTS for public health. The issue is further amplified by the emerging threat of multi‐drug resistant (MDR) *Salmonella*, as though the disease is usually self‐limiting, antimicrobial therapy is commonly necessary for invasive infections and recommended for patients at increased risk (Shane et al., 2017). Moreover, MDR NTS strains can act as reservoirs of resistance genes that can be horizontally transferred to other bacteria of the same or different species via mobile genetic elements, therefore contributing to continued spread of antimicrobial resistance (FAO, 2016).

According to recent source attribution studies, poultry, especially laying hens but also broilers and turkeys, is considered one of the major sources of foodborne salmonellosis (De Knegt, Pires, & Hald, 2015; Glass et al., 2016; IFSAC, 2015; Pires, Vieira, Hald, & Cole, 2014). Although *Salmonella* contamination is frequent in poultry production, predominant serotypes may change depending on host species, time period, region or production system. In response, European Union (EU) regulations have set targets for the reduction of specific serotypes, selected due to their public health significance in flocks of breeding hens, laying hens, broilers, breeding turkeys and fattening turkeys (Messens et al., 2013) (i.e. *S*. Enteritidis and *S*. Typhimurium, including its monophasic variant, for all species, plus *S*. Hadar, *S*. Virchow and *S*. Infantis in breeding hens). However, *Salmonella* infection in poultry (and other animal reservoirs) is known to show considerable variation, with frequent shifts in predominant serotypes (Foley, Lynne, & Nayak, 2008; Shah, Paul, Sischo, Crespo, & Guard, 2017) often driven by the expansion of highly dominant clones, such as *S*. Typhimurium phage type DT104 (Glynn et al., 1998; Threlfall, 2000). Still, the mechanisms by which such clones become dominant or why their frequency eventually decreases are not well understood (Branchu, Bawn, & Kingsley, 2018).

In Spain, *Salmonella* monitoring and control programmes in poultry are regulated by EU and Spanish laws and are implemented to ensure that all poultry populations reach the specific prevalence targets set for serotypes of public health relevance. Monitoring involves periodical testing of breeding flocks of *Gallus gallus* (every two weeks), flocks of laying hens (every 15 weeks) and flocks of broilers and turkeys (birds leaving for slaughter) as a responsibility of the food business operators (auto‐control checks) (Anon., 2003). In addition, the official veterinary services perform annual controls (official controls) on commercial farms depending on farm size (Anon., 2010, 2011, 2012a, 2012b). All the *Salmonella* isolates coming from official and a proportion of those originating from auto‐control checks (which are used when isolates from official checks do not reach the minimum required number) are then subjected to antimicrobial susceptibility testing, again following EU rules, to monitor the occurrence of antimicrobial resistance (AMR) in zoonotic agents (Anon., 2013). This allows the identification of trends of AMR occurrence and the comparison with trends in isolates recovered from food and human samples at a European and national level, typically expressed as changes of the aggregated proportion of resistant isolates to a given antimicrobial over time (EFSA & ECDC, 2018). However, analysis of this information at an isolate level can also help to identify multi‐resistance patterns, detect the spread of possible emerging strains and identify possible factors associated with their expansion (Aerts & Jaspers, 2012). The increasing availability of information and analytic approaches facilitated by the revolution in the field of information technology opens new possibilities for surveillance of infectious diseases and detection of emerging threats, including specific *Salmonella* clones (Besser, 2018).

In the present study, isolate‐level data from the Spanish national surveillance programme on AMR of *Salmonella* from poultry (laying hen, broiler and turkey) during 2011–2017 were analysed to assess the diversity in phenotypic resistance and to detect multi‐resistance patterns and emerging strains using univariate and multivariate analytic methods, including multiple correspondence analysis and hierarchical clustering.

## 2 MATERIAL AND METHODS

### 2.1 Study population and laboratory methods

All isolates included in this study were retrieved through official and auto‐control checks performed in flocks of laying hens (adults), broilers (within three weeks before slaughter) and fattening turkeys (within three weeks before slaughter) during 2011–2017 in Spain according to Commission Regulations No 517/2011, 200/2012 and 1190/2012 respectively (Anon, 2011, 2012a, 2012b). Isolates retrieved each year from any given host originated from independent epidemiological units (i.e. different flocks).

Faecal samples were analysed according to ISO 6579:2002/Amd1:2007 for samples collected from 2011 to 2016 and according to ISO 6579–1:2017 for 2017 samples. Further characterization of *Salmonella* isolates was performed by antimicrobial susceptibility testing. The following antimicrobials, tested against for all isolates in our database, were considered in this study: ampicillin (Amp), ciprofloxacin (Cip), nalidixic acid (Nal), chloramphenicol (Chl), gentamicin (Gen), sulfamethoxazole (Smx), tetracycline (Tet), trimethoprim (Tmp) and colistin (Cst). Minimal inhibitory concentrations (MIC) were determined using the two‐fold broth microdilution reference method, according to ISO 20776–1:2006. Interpretation of quantitative data was performed using ECOFF breakpoints indicated by the European Committee on Antimicrobial Susceptibility Testing (EUCAST) (EUCAST, 2019).

### 2.2 Statistical analysis

#### 2.2.1 Descriptive analysis

The proportion of isolates belonging to different serotypes and exhibiting specific resistance phenotypes was compared within/between host species using chi‐square tests corrected for multiple comparisons using Holm's method (Holm, 1979). The number of antimicrobials to which an isolate was resistant for each host species was compared using the Kruskal–Wallis test followed by the Dunn's post hoc test correcting for multiple comparisons through the Holm's method. In addition, information on the phenotypic resistance and yearly frequency of isolation of the most abundant serotypes (*n* > 200) were evaluated individually. Analyses were carried out in R (R core Team 2014) using the package FSA (Ogle, Wheeler, & Dinno, 2018).

#### 2.2.2 Analysis of diversity

The resistotype of each isolate was defined as the concatenation of its phenotype (resistant/non‐resistant) for each of the nine antimicrobials included in the study. Similarity between resistotypes present in isolates from each host species was evaluated using Venn diagrams, and within host species resistotype diversity distribution was evaluated using Simpson's Index of diversity D as follows (Hunter & Gaston, 1988):

$$D=1-\frac{\sum_{j=1}^{S}n_{j}\left(n_{j}-1\right)}{N\left(N-1\right)}$$

where *s* is the total number of resistotypes, $n_{j}$ is the number of isolates belonging to resistotype *j*, and *N* is the total number of isolates for each host. This index expresses the probability that two isolates that are randomly selected among the collection from a given host species will have different serotype/resistotype. Confidence intervals for the Simpson's indexes were estimated through 1,000 bootstrap replicates. Additionally, a rarefaction analysis to estimate the resistotype richness controlling for the different sample size in each host species was carried out as previously described (Ahlstrom et al., 2017). For serotypes in which at least 15 isolates from each host were recovered, the proportional similarity index (PSI), as described by Muellner et al. (2010), was used to compare resistotypes in each host. The PSI is an objective and simple measure of the area of intersection between two frequency distributions. It estimates the similarity between the frequency distributions of, for instance, bacterial subtypes (here resistotypes in a given serotype) from different sources. The value for the PSI ranges from 1 for identical frequency distributions to 0 for distributions with no common types, and bootstrap confidence intervals were estimated. Analyses were carried out in R (R core Team, 2014) using the Packages VEGAN and Venn.Diagram.

#### 2.2.3 Association between phenotypic resistances

For the most abundant serotypes (*n* > 50 isolates) the existence of associations between the simultaneous phenotypic resistance to any pair of antimicrobials was evaluated through Fisher's exact test using Holm's correction to adjust for multiple comparisons as previously described (Boerlin et al., 2005).

Then, a multiple correspondence analysis (MCA) was performed to analyse the multivariate pattern of relationship between the phenotypic resistances while accounting also for other nominal variables (here host species and serotype) and reduce the dimensions of the data as previously performed (Jaspers, Ganyani, Ensoy, Faes, & Aerts, 2016; Pages‐Monteiro et al., 2017). Clusters in the reduced dimensions obtained from the MCA, determined based on Euclidean distances, were investigated using hierarchical clustering with Ward's minimum variance method (Ward, 1963). The number of clusters was set empirically as described in Husson, Josse, & Pages (2010). Analyses were carried out using the R package FactoMineR (Le, Josse, & Husson, 2008).

## 3 RESULTS

### 3.1 Descriptive results

A total of 3,047 isolates from poultry were recovered during 2011–2017, of which 1,222 (40.1%) were retrieved from turkey samples, 1,060 (34.8%) from laying hens and 765 (25.1%) from broilers. The number of isolates recovered from each of the host species per year ranged between 26 and 226, with at least 110 isolates collected for each host‐year combination (except for broiler isolates recovered in years 2011–2013) (Table S1). Most of the isolates from laying hens were retrieved from official controls (82.7%) while for broiler and turkey the main source of isolates were auto‐controls performed by the food business operators (60.6 and 77.9% of the isolates, respectively), with large differences depending on the year (Table S1).

Overall, a total of 127 serotypes were identified, with the distribution of the main serotypes varying largely with the host (Table S2). In laying hens, no serotype accounted for more than 25% of the isolates, and serotypes other than *S*. Enteritidis (20.3%), *S*. Infantis (11.7%), *S*. Corvallis (8.8%) and *S*. Ohio (7.9%) represented less than 5% of the isolates. In total, those four most frequent serotypes accounted for 48.7% of all isolates from laying hens. In broilers, only six serotypes accounted for over 5% of the *Salmonella* isolates in this host, and jointly made up 73% of all isolates (*S*. Mikawasima, 16.1%; *S*. Kentucky, 14.4%; *S*. Virchow, 8.2%; *S*. Infantis, 7.2%; *S*. Havana, 5.9%; and *S*. Typhimurium, 5.1%). In the case of turkey, the top four serotypes (*S*. Derby, 51.4%; *S*. Hadar, 13.1%; *S*. London, 11.1%; and *S*. Kentucky, 6.4%) accounted for over 80% of the total with over half of the turkey isolates belonging to the most common serotype, *S*. Derby.

Frequency of phenotypic resistance was also highly dependent on the host species, with the proportion of isolates from laying hens resistant to any antimicrobial remaining below 20% (and below 11% except for the quinolones) compared with resistance rates above 20% for six and seven of the nine antimicrobials evaluated in the case of broiler and turkey isolates, respectively (Table 1). Significant host‐specific differences in the proportion of resistant isolates were identified for all antimicrobials, with the most common pattern being turkey > broiler>laying hen (for Amp, Chl, Cip, Smx, Tet and Tmp) followed by broiler > turkey>laying hen for Gen and Nal. The only antimicrobial in which the highest proportion of resistance was found for laying hens was Cst (Table 1). This was reflected by statistically significant (*p* < .0001) differences in the mean number of antimicrobials to which an isolate was resistant depending on the species, which was significantly (*p* < .0001) higher in the case of turkey (median = 5) compared with broiler (median = 2) and laying hen (median = 0), with the latter difference also being significant (*p* < .0001).

**Table 1:** Proportion of *Salmonella* isolates recovered from each host species resistant to the nine antimicrobials included in the study

Antimicrobial (ECOFF mg/L)	Total (*n* = 3,047)	Percentage of resistant isolates (>ECOFF)		
		Laying hen (*n* = 1,061)	Broiler (*n* = 765)	Turkey (*n* = 1,221)
Ciprofloxacin (0.064)	54.3	17.1^a^	61.3^b^	**82.2** ^c^
Ampicillin (8)	45.5	6.03^a^	36.5^b^	**85.4** ^c^
Tetracycline (8)	43.0	10.5^a^	23.8^b^	**83.2** ^c^
Sulfamethoxazol (256)	40.9	7.54^a^	32.0^b^	**75.3** ^c^
Trimethoprim (2)	27.3	2.64^a^	7.32^b^	**61.4** ^c^
Nalidixic acid (16)	26.5	14.3 ^a^	**39.7** ^b^	28.7^b^
Chloramphenicol (16)	17.0	1.41^a^	5.23^b^	**37.8** ^c^
Gentamycin (2)	8.66	1.70^a^	**20.1** ^b^	7.53^c^
Colistin (2)	2.98	**6.41** ^a^	1.44^b^	0.98^b^

Note

Different superscripts indicate significant differences in the proportion of resistant isolates between hosts; the host with the highest proportion of resistance to each antimicrobial is indicated in bold

### 3.2 Diversity of AMR phenotypes

**Figure 1:**
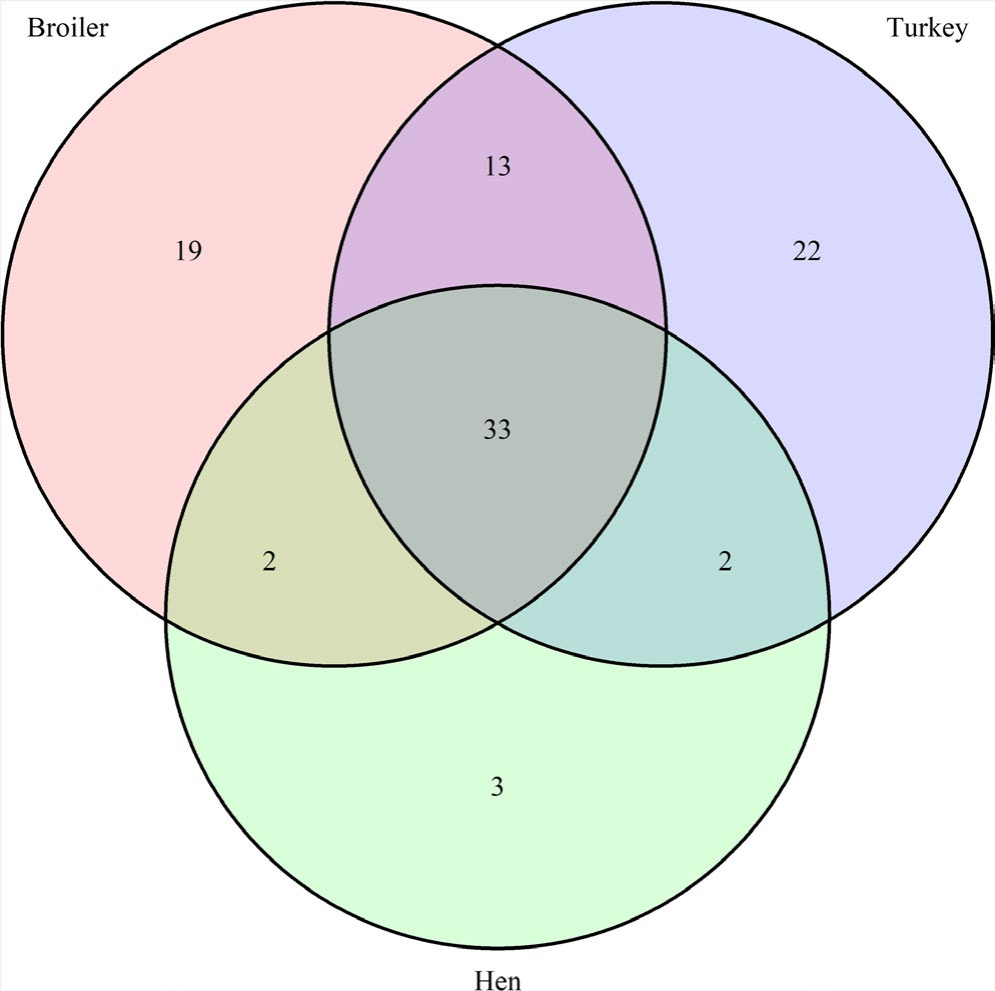
Venn diagram showing the degree of overlap of 94 resistotypes present in 3,047 *Salmonella* isolates retrieved from laying hen, broiler and turkey samples in 2011–2017 in Spain [Colour figure can be viewed at http://www.wileyonlinelibrary.com/]

**Figure 2:**
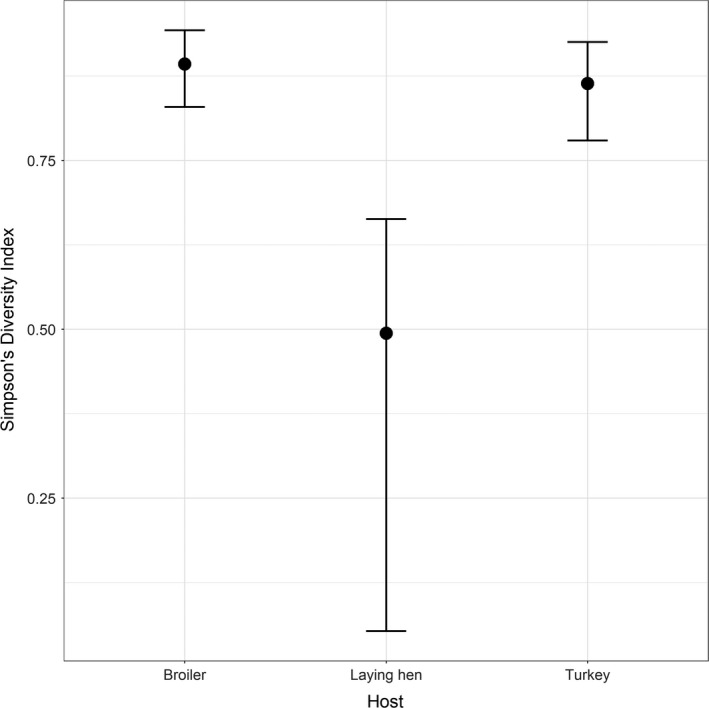
Diversity in the distribution of resistotypes depending on the host of origin in 3,047 *Salmonella* isolates retrieved in 2011–2017 in Spain. Bars represent confidence intervals around the Simpson's diversity index estimated through 1,000 bootstrap replicates

**Figure 3:**
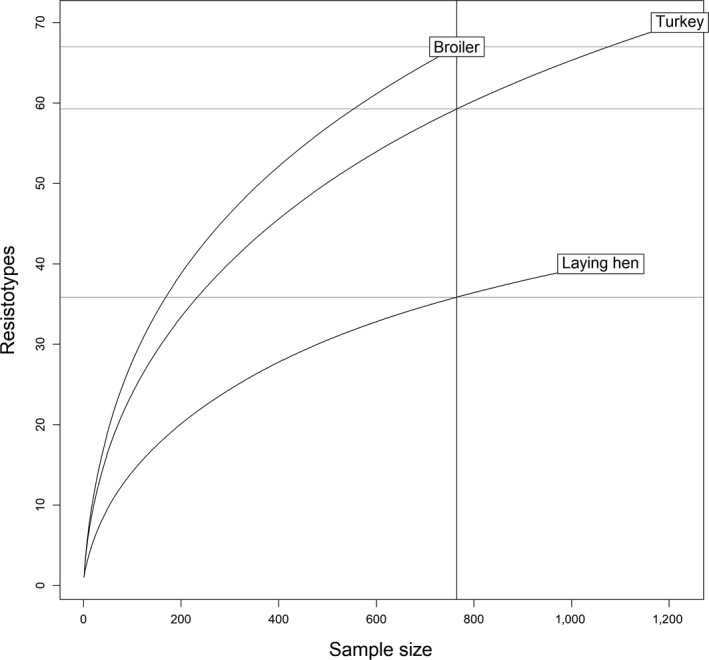
Resistotype richness in 3,047 *Salmonella* isolates retrieved from laying hen, broiler and turkey samples in Spain in 2011–2017. Change in resistotype accumulation curves with increasing sample sizes for isolates recovered from samples of broiler, turkey and laying hen

Overall, 94 out of the total 2^9 = 512 possible resistotypes were found in the isolate collection, of which 67 were found among broiler isolates, 70 among turkey isolates and 40 among laying hen isolates; approximately one‐third of the resistotypes (33/94) were present in isolates from all three hosts, while 22, 19 and 3 were exclusive to broiler, turkey and laying hen isolates, respectively (Figure 1). Resistotype diversity was similar for isolates from broiler (Simpson's Index = 0.89, 95% confidence interval 0.83–0.94) and turkey (0.86, 95% CI 0.78–0.93), and higher than that for isolates from laying hen (0.49, 95% CI 0.05–0.65) (Figure 2). These results were in agreement with the rarefaction analysis: the resistotype accumulation curves for broiler and turkey showed considerably steeper slopes so that an increasing sample size was translated into a more drastic increase of resistotypes compared with laying hen, thus pointing out a larger richness of resistotypes in turkey and broiler isolates compared with those from laying hen (Figure 3).

A serotype‐specific PSI was only calculated for four serotypes in which > 15 isolates from each host were retrieved (i.e. Kentucky, Mikawasima, Senftenberg and Typhimurium). Proportional similarity between resistotypes was highly dependent on the serotype: for *S*. Mikawasima isolates, there was a very high similarity between resistotypes from different hosts (>0.85 for all comparisons, with one highly dominant resistotype observed) while on the other hand, for *S*. Senftenberg the similarity observed was consistently low (<0.25) (Supporting Information Table S3). Interestingly, *S*. Typhimurium resistotypes from broilers were significantly less similar to those from laying hens (0.14; 95% CI 0–0.34) than to turkey isolates. A similar pattern, but not significant, could be observed for *S*. Kentucky (Table S3).

### 3.3 Association between phenotypic resistances

Bivariate association between phenotypic resistance against any two antimicrobials was assessed in 15 serotypes with *n* > 50 (Table S4). The most common significant associations across serotypes were found for the two quinolones tested, Cip‐Nal (significantly associated with 11/15 serotypes). Regarding antimicrobials from different families, the most common pattern of resistance involved Amp‐Tet, Cip‐Tet and Smx‐Tet (associated with 7/15 serotypes). A high (>25) number of significant associations with other antimicrobials across serotypes was found for six out of all nine antimicrobials considered, while no significant association was found for Cst and few for Chl (*n* = 17) and Gen (12) (Table S4). Per serotype, the highest number of associations between different antimicrobials was found for Agona (17/36 possible pairs), Kentucky (16/36), Typhimurium (15/36) and London (14/36). On the other end, less than five significantly associated pairs of antimicrobials were found for Corvallis (0), Havana, Monophasic and Ohio (1), Mikawasima (3) and Enteritidis and Virchow (4) (results not shown).

**Figure 4:**
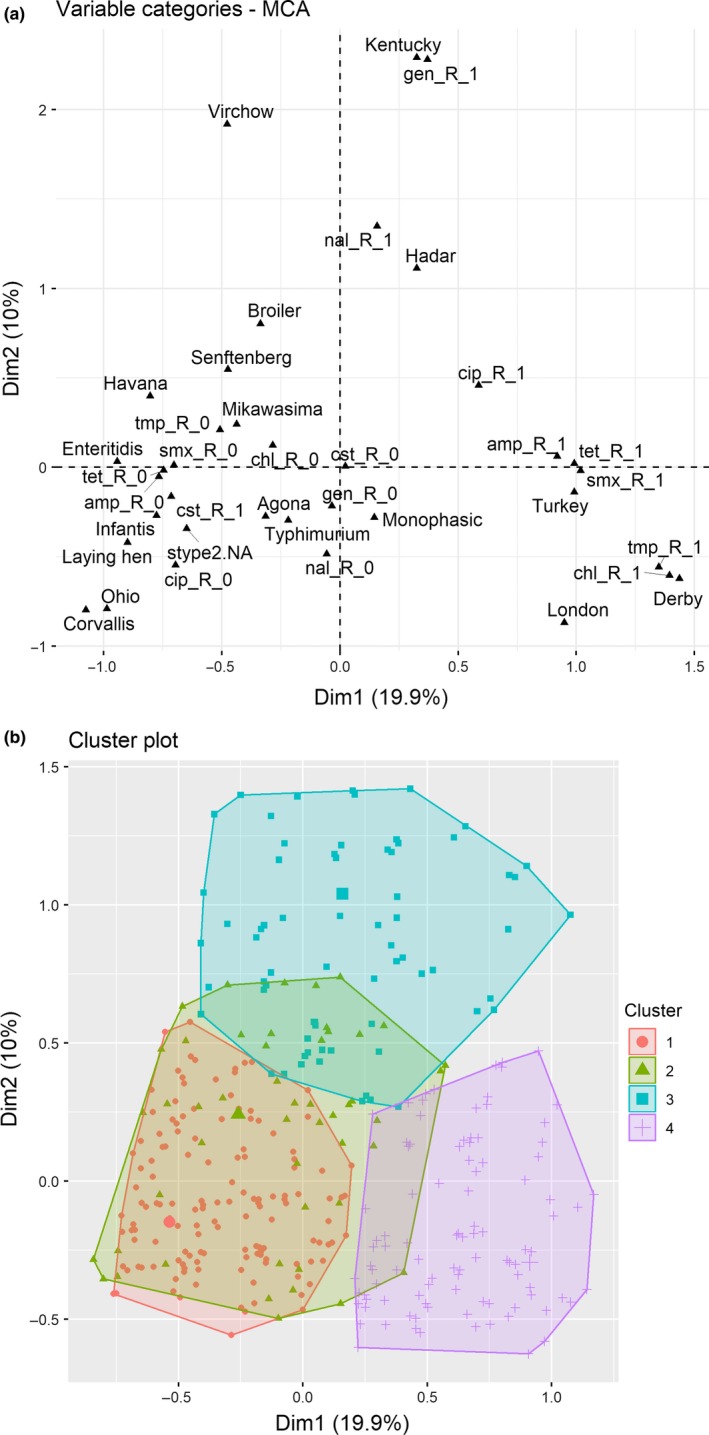
Multiple correspondence analysis (MCA) of 3,047 *Salmonella* isolates retrieved in 2011–2017 in Spain (first two dimensions). (a) Distribution of the antimicrobial resistance, serotype and host variables included in the MCA. Antimicrobial resistance variables are indicated by the abbreviation followed by R_1 (resistance) or R_0 (susceptibility). (b) Distribution of observations into four clusters as determined by hierarchical clustering [Colour figure can be viewed at http://www.wileyonlinelibrary.com/]

The first three dimensions identified in the MCA explained up to 36% of the total variability in the resistance phenotypes, host and serotype of the *Salmonella* collection. Variables most associated with these dimensions are shown in Table 2. While the first dimension (19.9% variability) was composed of positive contributions of isolates resistant to multiple antimicrobials, recovered from turkey and belonging primarily to the Derby and London serotypes, in the second dimension (10.0% variability) the serotypes with a higher contribution were Kentucky, Virchow and Hadar, the primary associated host species was broiler and the phenotypic resistances with the highest contribution were Nal, Gen and Cip (and being susceptible to Tmp and Chl). The third dimension, that explained only 6.3% of the variability, included Enteritidis and laying hens as the most associated serotype and host and was the only dimension to which resistance to Cst was found to be contributing significantly (Table 2). The graphical representation of the MCA for dimensions 1 and 2 shows the variables referring to resistance to Amp, Smx and Tet were clustered with the turkey origin, and close to the cluster containing resistance to Chl, Tmp and the Derby serotype (and resistance to Cip), while in the vertical axis resistance to Gen and, to a lesser extent, Nal, and the Kentucky serotype was clustered in high values for dimension 2 (and low values for dimension 1) (Figure 4a). The horizontal axis (dimension 1) represented in fact a gradient of resistance to the different antimicrobials, with susceptible results having negative values except in the case of Cst (Figure 4a). Four clusters were identified in the hierarchical clustering analysis performed on the first three dimensions of the MCA (Figure 4b). Cluster 1 included approximately 45% of all isolates (1,380/3,047) that were mostly originated from laying hen (778/1,380, including ~ 73% of all laying hen isolates) and broiler samples (465/1,380, and ~ 61% of all broiler isolates). Cluster 4 contained approximately 29% of the collection (878/3,047) and was mostly formed by turkey isolates (825/878, and ~ 68% of all turkey isolates). Clusters 2 and 3 were smaller (445 and 344 isolates, respectively): cluster 2 included predominantly a combination of isolates from laying hen (238/445 (53.5%), of which most (216/238) were Enteritidis) and turkey (167/445 (37.5%), of which most (152/167) were Hadar). Cluster 3, in turn, was formed by broiler and turkey isolates (65.4% and 26.7%, respectively), that belonged predominantly to the Kentucky serotype (210/344) and in fact included most Kentucky isolates (210 out of a total of 228 isolates; 92%).

**Table 2:** Description of the three first dimensions identified in a multiple correspondence analysis performed on the host, serotype and resistance phenotype to nine antimicrobials of 3,047 *Salmonella* isolates recovered from poultry

Dimension 1 (19.9%)		Dimension 2 (10.0%)		Dimension 3 (6.2%)	
Variable	Estimate (*p*‐value)	Variable	Estimate (*p*‐value)	Variable	Estimate (*p*‐value)
Derby	1.16 (<.0001)	Kentucky	1.05 (<.0001)	Enteritidis	0.93 (<.0001)
Turkey	0.73 (<.0001)	Nal‐R	0.44 (<.0001)	Cst‐R	0.63 (<.0001)
Tmp‐R	0.64 (<.0001)	Gen‐R	0.61 (<.0001)	Hadar	0.74 (<.0001)
Tet‐R	0.60 (<.0001)	Cip‐R	0.24 (<.0001)	Nal‐R	0.16 (<.0001)
Smx‐R	0.59 (<.0001)	Broiler	0.35 (<.0001)	Gen‐S	0.20 (<.0001)
Cip‐R	0.44 (<.0001)	Tmp‐S	0.19 (<.0001)	Laying hen	0.16 (<.0001)
Chl‐R	0.58 (<.0001)	Virchow	0.87 (<.0001)	Cip‐R	0.08 (<.0001)
Amp‐R	0.58 (<.0001)	Hadar	0.48 (<.0001)	Smx‐S	0.07 (<.0001)
London	0.82 (<.0001)	Chl‐S	0.18 (<.0001)	Turkey	0.10 (<.0001)

### 3.4 Most frequent serotypes

#### 3.4.1 *Salmonella* Derby

**Figure 5:**
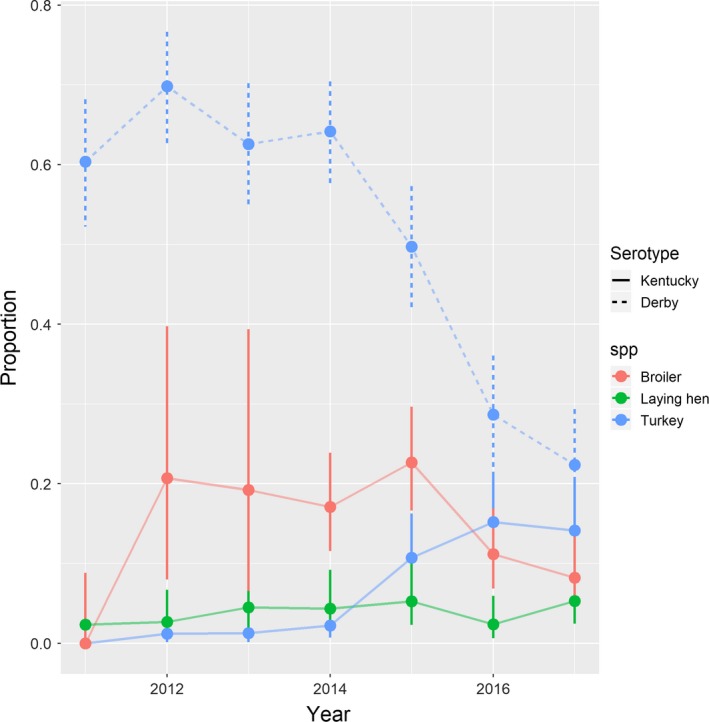
Proportion of isolates recovered from laying hen, broiler and turkey samples belonging to the *Salmonella* Kentucky and *Salmonella* Derby (only turkey isolates) serotypes during 2011–2017. Bars represent confidence intervals around the proportions estimated using the Pearson‐Klopper method [Colour figure can be viewed at http://www.wileyonlinelibrary.com/]

The most common serotype in the collection in this study was *S.* Derby (637/3,047; 21% of all isolates), with almost all isolates recovered from turkey (628/637) and only four and five isolates cultured from broiler and laying hen samples, respectively. As discussed above, more than 50% of the turkey isolates were identified as *S.* Derby, although the proportion of turkey isolates belonging to this serotype in our collection decreased sharply during the study period, dropping from > 60% in 2011–2014 to less than 30% in 2017 (Figure 5).

Resistance to Amp, Cip, Smx, Tet and Tmp was extremely high (>89%) among *S.* Derby isolates, while resistance to Chl was high (46.3%, 295/637). In contrast, resistance to Cst, Gen and Nal was very low to low (0.7, 0.3 and 8.9% respectively). Out of the 572 Cip‐Resistant (Cip‐R) *S.* Derby isolates found, only 57 (10.0%) were also resistant to Nal, and thus, 90% of the isolates displayed the ‘nontypical’ quinolone resistance phenotype (Cip‐R/Nal‐S) (Gunell et al., 2009).

Most *S*. Derby isolates (627/637, 98.4%) belonged to Cluster 4, with another seven isolates (susceptible to all antimicrobials except for Smx (*n* = 2) and Tet (*n* = 5)) allocated to Cluster 1 and the remaining two isolates classified in Clusters 2 and 3.

#### 3.4.2 *Salmonella* Enteritidis

A total of 238 *S*. Enteritidis isolates were included in the panel during the study period, mostly from laying hen (216/238; 91%) with the remainder being recovered from broiler samples. The proportion of all laying hen isolates identified as *S.* Enteritidis was higher during the two first years of study (34.7% and 28.7% in 2011 and 2012, respectively) than in the remaining period (between 10% and 18%), while recovery of isolates from this serotype from broiler samples was always a rare event (less than eight isolates recovered in any given year).

Resistance was low‐to‐very low (<3%) for most antimicrobials except Nal and Cip (high levels of resistance, 48.7%; 116/238, with all isolates being resistant to both antimicrobials simultaneously) and Cst (moderate resistance, 28.2%, 67/238). A significantly (*p* = .0097) higher proportion of Nal‐Cip‐resistant isolates was found among broiler isolates despite the small sample size (17/22) compared to laying hen (99/216), while no differences in the proportion of Cst‐resistant isolates were observed (4/22 vs. 63/216, *p* = .4).

The majority of the *S*. Enteritidis isolates were classified in Cluster 2 (233/238), with the only exception being five fully susceptible isolates recovered from broiler that were allocated to Cluster 1.

#### 3.4.3 *Salmonella* Kentucky

Overall 228 isolates identified as *S*. Kentucky were retrieved over the study period, of which 110 were isolated from broiler (48%), 40 from laying hen (18%) and 78 from turkey (34%). The proportion of isolates from turkey belonging to this serotype in the collection increased dramatically over the study period, going from less than 3% before 2015 to 18.5% in 2016–2017, while no similar trend during the whole study period was observed in the case of isolates from laying hen or broilers (Figure 5).

The resistance profile of the *S*. Kentucky isolates appeared to depend highly on the host, with a median number of 2.5, 5 and 6 resistances to different antibiotics in isolates from laying hen, broiler and turkey, respectively. Thirty per cent of the laying hen isolates were fully susceptible, whereas the most common resistotype in the isolates from the other two hosts included co‐resistance to Amp, Cip, Gen, Nal, Smx and Tet, with up to 30% and 60% of the broiler and turkey isolates respectively presenting this pattern (Table 3). Overall, the proportion of isolates that were resistant to these six antimicrobials (with or without additional resistances) was high (43.0%, 98/228), and this pattern of resistance was particularly common among turkey isolates (57/78, 73.1%) compared to broiler (36/110, 32.7%) and laying hen isolates (5/40, 12.5%). Isolates with this resistance profile were first observed in 2011 in an isolate from a laying hen, in 2012 from turkey and in 2014 from broiler. However, its frequency over time was different depending on the host species; for example, it was always predominant among turkey isolates even when few *S*. Kentucky were retrieved from this host species (6/9 in 2011–2014 and 51/69 in 2015–2017) and always uncommon among the few *S*. Kentucky recovered from laying hens (3/19 in 2011–2014 and 2/21 in 2015–2017). In the case of broiler, however, its frequency increased over time (0/11 in 2011–2013, 20/66 in 2014–2015 and 16/33 in 2016–2017).

**Table 3:** Number and percentage of antimicrobial‐resistant phenotypes for 228 *Salmonella* Kentucky isolates per host on the basis of the number of antimicrobials to which the isolates were resistant. The most common resistotype in each host and overall is indicated in bold

Number of antibiotics	Resistotype	Laying hen	Broiler	Turkey	% of isolates (number)
0	Pansusceptible	**12 (30.0)**	2 (1.81)	1 (1.28)	15 (6.57)
1	Tet | Smx^a^	0 (0)	2 (1.81)	0 (0)	2 (0.87)
2	Cip, Nal	7 (17.5)	8 (7.27)	2 (2.56)	17 (7.45)
	Others	1 (2.50)	0 (0)	2 (2.56)	3 (1.31)
3	Cip, Nal, Amp	0 (0)	18 (16.3)	3 (3.84)	21 (9.21)
	Cip, Nal, Tet	5 (12.5)	0 (0)	0 (0)	5 (2.19)
	Cip, Nal, Gen	0 (0)	4 (3.63)	1 (1.28)	5 (2.19)
	Cip, Amp, Chl	0 (0)	1 (0.90)	0 (0)	1 (0.43)
4	Cip, Nal, Gen, Smx	1 (2.50)	11 (10.0)	3 (3.84)	15 (6.57)
	Cip, Nal, Amp, Tet	0 (0)	1 (0.90)	0 (0)	1 (0.43)
5	Cip, Nal, Gen, Amp, Smx	2 (5.00)	17 (15.4)	3 (3.84)	22 (9.64)
	Cip, Nal, Gen, Smx, Tet	7 (17.5)	8 (7.27)	6 (7.69)	21 (9.21)
	Others	0 (0)	2 (1.81)	0 (0)	2 (0.87)
6	Cip, Nal, Gen, Amp, Smx, Tet	4 (10.0)	**33 (30.0)**	**47 (60.2)**	**84 (36.8)**
7	Cip, Nal, Gen, Amp, Smx, Tet, Chl	0 (0)	2 (1.81)	2 (2.56)	4 (1.75)
	Cip, Nal, Gen, Amp, Smx, Tet, Tmp	0 (0)	0 (0)	2 (2.56)	2 (0.87)
8	Cip, Nal, Gen, Amp, Smx, Tet, Tmp, Chl,	1 (2.50)	1 (0.90)	6 (7.69)	8 (3.50)
	Total	40 (100)	110 (100)	78 (100)	228 (100)

a Resistant to either Tet or Smx.

The majority of *S*. Kentucky isolates (210/228, 92.1%) were assigned to Cluster 3, with the remaining 18 isolates (13 from laying hen, 4 from broiler and one from turkey) assigned to Cluster 1 and showing full susceptibility except for two isolates resistant alone to Smx and Tet.

## 4 DISCUSSION

Given the importance of poultry and poultry products as sources of foodborne salmonellosis, national control programmes are a critical tool to ensure that presence of *Salmonella* remains at minimum levels in poultry populations, particularly for serotypes of special importance to public health (Messens et al., 2013). In addition, these programmes enable the identification of predominant serotypes and AMR phenotypes that can be used to better understand the epidemiology of *Salmonella* infection in the food animal reservoir. Here, we have demonstrated that data from the Spanish control programme in poultry can be used to provide valuable insight into *Salmonella* population dynamics in laying hens, turkey and broiler that could be indicative of expanding clones or trends in serotype or resistotype dominance.

In our collection, serotype distribution was much more heterogeneous in isolates from laying hen compared with broiler and especially turkey, from which half of the isolates recovered belonged to a single serotype that nevertheless became less frequent at the end of the study period. Care should be taken however when interpreting serotype data from broiler and turkeys since over half of the isolates included in this study for those two hosts were selected through convenience sampling among isolates retrieved during the controls performed by the food business operators ensuring only they originated from different epidemiological units, and may therefore not represent the true distribution of serotypes in these poultry populations. Therefore the shifts observed in serotypes over time for broiler and turkey isolates may be partially due to sampling bias and not represent a true change in their prevalence. Still, a comparison of the ranks in the proportion of isolates belonging to each serotype found in auto‐control checks performed by food business operators with those found in this study revealed no significant differences, thus suggesting that results approximate the field situation (results not shown).

While the rarefaction analysis suggested that a large amount of the resistotype diversity in *Salmonella* isolates circulating in laying hens was being captured through the achieved sample size, this was not the case for isolates from broiler and turkey even if they were more homogeneous in terms of serotype diversity. This is likely a result of the higher proportion of susceptible isolates to all the antimicrobials except Cst recovered from laying hen compared with those from turkey or broiler, which could reflect a higher use of antimicrobials in the latter two hosts (70.3% of all isolates from laying hens were pansusceptible). Interestingly, among the four serotypes in which at least 15 isolates from all three host types were found, only one (*S*. Mikawasima) had a highly similar resistotype regardless of the host species, which could be explained by a frequent resistotype (Amp‐Cip) found in all three hosts. For the remaining three serotypes (Senftenberg, Kentucky and Typhimurium), isolates from laying hens were always less similar, therefore suggesting that in certain cases the production environment (including antimicrobial use) could play a major role in resistotype occurrence within a serotype versus resistance being mainly determined by the serotype alone.

Colistin was the antimicrobial against which the proportion of resistant isolates was lowest in all three host types. Most (67/91) of the Cst‐resistant isolates were identified as *S*. Enteritidis, which is in agreement with previous reports from other European countries, suggesting that this phenotype may be associated with intrinsic serotype‐specific differences in susceptibility (Agerso et al., 2012; EFSA & ECDC, 2018). The presence of several plasmid‐mediated Cst‐resistance genes (*mcr‐1*, *mcr‐2, mcr‐4, mcr‐5* and *mcr‐9)* in *Salmonella* has previously been reported in isolates with typically high levels of phenotypic/genotypic resistance to a large number of antimicrobials (Borowiak et al., 2017; Carattoli et al., 2017; Carroll et al., 2019; Garcia‐Graells et al., 2018; Hu et al., 2019; Skov & Monnet, 2016). The fact that most (>75%) of the Cst‐resistant isolates found in this study were in contrast susceptible to all other antimicrobials tested except quinolones suggests that the plasmid‐mediated *mcr* genes may not have been a frequent source of Cst‐resistance in the Spanish isolates. This is in agreement with previous results obtained in NTS isolates retrieved from poultry in Spain in 2010–2017 with a suspected (MIC = 2) or confirmed (MIC ≥ 4) Cst‐resistant phenotype screened for the presence of *mcr*‐1 and *mcr*‐2 (*n* = 37) or *mcr*‐1 to *mcr*‐5 (*n* = 115) using PCR. In this analysis, *mcr* genes were only detected in four (all *mcr‐1*) and six (five *mcr‐1*, one *mcr‐4*) isolates, respectively (Agüero M., personal communication).

Nevertheless, MDR Cst‐resistant strains were also found in low frequencies here, including four *S*. Derby and two *S*. London isolates resistant to > 5/8 antimicrobials evaluated in addition to Cst, similar to what has been described for *mcr‐1*‐positive *Salmonella* isolates belonging to these serotypes retrieved from food in China (Hu et al., 2019). Therefore, additional genomic characterization studies of *S*. Enteritidis isolates but also of MDR strains belonging to other serotypes would be required in order to assess the role of plasmid‐mediated genes in the development of Cst‐resistance in *Salmonella* isolates of poultry origin in Spain.

Bivariate and multivariate analyses confirmed the significant association between the occurrence of simultaneous phenotypic resistance to several antimicrobials, particularly Amp‐Tet‐Smx, across most serotypes, with > 70% of all isolates resistant to either Amp, Tet or Smx exhibiting simultaneous resistance to the other two antimicrobials. Moreover, 79.2% and 46.7% of all Amp‐Tet‐Smx resistant isolates (*n* = 967) were also resistant to Tmp or Chl, respectively. This would be compatible with the presence of variants of the *Salmonella* genomic island (SGI) 1 (Levings, Djordjevic, & Hall, 2008), which was first identified in the epidemic DT104 *S.* Typhimurium clone but that based on the phenotypes observed here would be particularly common in other serotypes (with > 45% of all *S.* Derby and *S.* London isolates exhibiting a Amp‐Chl‐Smx‐Tet‐Tmp resistance profile).

Resistance to Cip and Nal were also significantly associated with most prevalent serotypes (Table S4) as expected given that they both belong to the same antimicrobial family, i.e. quinolones. Still, in certain serotypes (e.g. Derby, London and Mikawasima) in which the proportion of Cip‐resistant isolates was > 50% the observed pattern was actually resistance to Cip and susceptibility to Nal. This non‐typical quinolone resistance phenotype, reported in poultry isolates from several member states (EFSA & ECDC, 2018), is usually observed in strains with no mutations in the quinolone resistance‐determining regions but rather in strains that harbour plasmid‐mediated quinolone resistance (PMQR) genes (often from the *qnr* family) (Gunell et al., 2009). The emergence of *Salmonella* strains circulating in livestock and carrying PMQR genes has been recently reported in several regions of the world (Elnekave et al., 2019, 2018; Lin, Chen, Wai‐Chi Chan, & Chen, 2015) and represents a major public health concern due to their ability to be transferred horizontally.

The existence of detectable clusters in the distribution of the isolates across the first three dimensions identified in the MCA further confirmed the association between groups of isolates based on the information on their AMR phenotype, serotype and host species. While Cluster 1 included the majority of the (susceptible) broiler and laying hen isolates, Cluster 4 included most turkey isolates that were also identified as *S*. Derby, the most prevalent serotype in the panel, and presented a MDR phenotype. Previous studies have suggested that this serotype, commonly found in pig and poultry, is polyphyletic, with certain lineages being primarily recovered from either pig or poultry (Hayward, Petrovska, Jansen, & Woodward, 2016; Sevellec et al., 2018). Interestingly, typing of 140 isolates representative of the pork and poultry food sectors in France retrieved in 2014–2015 revealed that while MDR phenotypes (with resistance to amynoglycosides, sulphonamides and tetracycline) were associated with pig‐specific lineages in France, isolates from poultry were typically pansusceptible (Sevellec et al., 2018). In fact, *S*. Derby isolates retrieved from poultry in 2016 in France and the UK as part of their AMR monitoring programmes showed lower levels of phenotypic resistance to all antimicrobials compared with Spanish isolates (EFSA & ECDC, 2018). Outbreak‐related *S*. Derby isolates of pork origin have been previously described in Spain both with typically resistant (Valdezate et al., 2005) and susceptible (Arnedo‐Pena et al., 2016) phenotypes. Additional studies would be required in order to assess the genetic variability among the Amp‐Cip‐Smx‐Tet‐Tmp resistant *S*. Derby turkey isolates found here and its genetic relatedness with pig strains.

Clusters 2 and 3 contained combinations of laying hen‐turkey and broiler‐turkey isolates and were dominated by specific serotype‐species combinations (Enteritidis‐laying hen and Hadar‐turkey for Cluster 2 and Kentucky in both broiler and turkey for Cluster 3). Results on serotype Kentucky were particularly interesting due to the global spread of a specific *S*. Kentucky MDR strain (ST198‐X1) with high‐level resistance to Cip (Le Hello, Bekhit, et al., 2013). This strain, initially recovered in Europe from travellers to African countries in the 2000’s (Le Hello et al., 2011), has since then been isolated from human and non‐human sources in Europe, Africa and Asia, resulting in major public health concerns due to the variant's carriage of multiple resistance genes including extended‐spectrum beta‐lactamase (ESBL), plasmid‐encoded cephalosporinase and carbapenemase (Le Hello, Bekhit, et al., 2013; Le Hello, Harrois, et al., 2013). In the most recent reports on AMR in zoonotic and indicator bacteria from humans, animals and food, *S*. Kentucky was the seventh/eight most common serotype recovered from humans in 2016/2017, but also displayed one of the highest levels of MDR (76.3% compared with a general level of 26.5% across all serotypes), and around 20% of the human *S*. Kentucky isolates carried ESBLs (EFSA & ECDC, 2018, 2019). Even though limited numbers of *S*. Kentucky from broiler (*n* = 79) and turkey (*n* = 46) flocks were also reported in 2016 from EU member states, most of them (>60% in broiler and > 80% in turkey) were resistant to Amp‐Cip‐Gen‐Nal‐Smx‐Tet (EFSA & ECDC, 2018). According to our data, spanning a 7‐year period, this MDR phenotype may be increasingly common in turkey and broiler flocks since 2015, although the potential biases associated with the non‐random selection of isolates from auto‐control checks make it necessary to confirm this finding. Given the high MICs observed in the *S*. Kentucky MDR isolates found in this study for Cip (>4 mg/L, data not shown), our results indicate that the ST198‐X1 epidemic strain may have become established in turkey and broiler flocks in Spain. Even though the risk to public health in the country at this point seems low (with no MDR *S*. Kentucky reported from Spain in 2016) (EFSA & ECDC, 2018), additional studies to evaluate the diversity among MDR *S*. Kentucky strains from poultry as well as factors associated with its establishment are much needed.

In summary, our study demonstrates the usefulness of the analysis of data from AMR monitoring programmes at the isolate level. Our results suggest that, while the sample size achieved over seven years captures only a proportion of the variability in AMR phenotypes in *Salmonella* circulating in poultry (especially in broiler and turkey flocks), certain clusters of resistance phenotypes are particularly common among turkey and broiler isolates from specific serotypes, which provide indication on the possible genetic background conferring different phenotypic resistance (i.e. SGI‐1 or PMQR) that should be confirmed using molecular characterization techniques. Furthermore, our results revealed a dynamic situation in terms of predominant serotypes, with MDR strains potentially being replaced by others (such as Derby by Kentucky in turkey). The information provided can be used to identify sources and factors associated with *Salmonella* spread in poultry and therefore implement measures to mitigate the considerable public health risk posed by this zoonotic bacterium.

## ETHICS STATEMENT

The authors confirm that the ethical policies of the journal, as noted in the journal's author guidelines page, have been adhered to. No ethical approval was required as all isolates analysed here were retrieved through the ongoing Spanish national surveillance program on AMR performed according to national and EU regulations.

## CONFLICT OF INTEREST

The authors declare no conflicts of interests in relation to this work.
