# Supplementary material for: Identifying emerging trends in antimicrobial resistance using Salmonella surveillance data in poultry in Spain
Source: Transbound Emerg Dis. 2019 Sep 13;67(1):250–62. doi: 10.1111/tbed.13346 (PMC7028142; doi:10.1111/tbed.13346)
Supplement: Supplementary file 1 [file TBED-67-250-s001.docx]

Table S1. Number and sources of *Salmonella* isolates recovered from each host over the 2011-2017 included in the study

| Year | Broiler | | | | Laying hen | | | | Turkey | | | | Official | FBO | NA | Total |
| --- | --- | --- | --- | --- | --- | --- | --- | --- | --- | --- | --- | --- | --- | --- | --- | --- |
| Source | Official | FBO† | NA‡ | Total | Official | FBO | NA | Total | Official | FBO | NA | Total |  |  |  |  |
| 2011 | 40 | 0 | 0 | 40 | 169 | 0 | 1 | 170 | 26 | 126 | 2 | 154 | 235 | 126 | 3 | 364 |
| 2012 | 26 | 0 | 3 | 29 | 119 | 1 | 30 | 150 | 151 | 3 | 15 | 169 | 296 | 4 | 48 | 348 |
| 2013 | 26 | 0 | 0 | 26 | 95 | 1 | 15 | 111 | 42 | 112 | 1 | 155 | 163 | 113 | 16 | 292 |
| 2014 | 61 | 97 | 0 | 158 | 118 | 20 | 0 | 138 | 18 | 208 | 0 | 226 | 197 | 325 | 0 | 522 |
| 2015 | 59 | 113 | 0 | 172 | 98 | 54 | 0 | 152 | 11 | 166 | 0 | 177 | 198 | 333 | 0 | 510 |
| 2016 | 31 | 138 | 1 | 170 | 111 | 58 | 0 | 169 | 9 | 162 | 0 | 171 | 151 | 358 | 1 | 510 |
| 2017 | 56 | 114 | 0 | 170 | 138 | 32 | 0 | 170 | 9 | 161 | 0 | 170 | 203 | 307 | 0 | 510 |
| Total | 299 | 462 | 4 | 765 | 848 | 166 | 46 | 1,060 | 266 | 938 | 18 | 1,222 | 1413 | 1566 | 68 | 3,047 |

†FBO: isolates retrieved in auto-controls performed by the food business operators

‡NA: Not available (data not recorded)
