# Supplementary material for: Identifying emerging trends in antimicrobial resistance using Salmonella surveillance data in poultry in Spain
Source: Transbound Emerg Dis. 2019 Sep 13;67(1):250–62. doi: 10.1111/tbed.13346 (PMC7028142; doi:10.1111/tbed.13346)
Supplement: Supplementary file 3 [file TBED-67-250-s003.docx]

Table S3. Proportional similarity index (with bootstrapped 95% confidence intervals) of the four serotypes with >15 isolates retrieved from all three hosts based on their resistance to nine antimicrobials.

| Serotype |  | Broiler | Laying hen |
| --- | --- | --- | --- |
| Mikawasima | Broiler | - | - |
|  | Laying hen | 0.94 (0.90-0.98) | - |
|  | Turkey | 0.91 (0.72-0.97) | 0.88 (0.71-1.00) |
| Senftenberg |  | Broiler | Laying hen |
|  | Broiler | - | - |
|  | Laying hen | 0.03 (0.00-0.10) | - |
|  | Turkey | 0.13 (0.03-0.26) | 0.20 (0.00-0.48) |
| Kentucky |  | Broiler | Laying hen |
|  | Broiler | - | - |
|  | Laying hen | 0.41 (0.24-0.56) | - |
|  | Turkey | 0.56 (0.42-0.66) | 0.36 (0.18-0.49) |
| Typhimurium |  | Broiler | Laying hen |
|  | Broiler | - | - |
|  | Laying hen | 0.14 (0.00-0.34) | - |
|  | Turkey | 0.73 (0.42-0.77) | 0.28 (0.04-0.47) |
