# Supplementary material for: Identifying emerging trends in antimicrobial resistance using Salmonella surveillance data in poultry in Spain
Source: Transbound Emerg Dis. 2019 Sep 13;67(1):250–62. doi: 10.1111/tbed.13346 (PMC7028142; doi:10.1111/tbed.13346)
Supplement: Supplementary file 4 [file TBED-67-250-s004.docx]

Table S4. Number of serotypes out of 15 with n>50 isolates retrieved from poultry in Spain during 2010-2017 in which a significant association between the simultaneous presentation of phenotypic resistance against a given pair of antimicrobials was found. Significance was determined using a Fisher’s exact test using Holm’s correction to adjust for multiple comparisons.

|  | Chl | Cip | Cst | Gen | Nal | Smx | Tet | Tmp |
| --- | --- | --- | --- | --- | --- | --- | --- | --- |
| Amp | 3 | 6 | 0 | 2 | 3 | 5 | 7 | 4 |
| Chl |  | 2 | 0 | 0 | 2 | 3 | 3 | 4 |
| Cip |  |  | 0 | 2 | 11 | 6 | 7 | 4 |
| Cst |  |  |  | 0 | 0 | 0 | 0 | 0 |
| Gen |  |  |  |  | 3 | 3 | 2 | 0 |
| Nal |  |  |  |  |  | 3 | 5 | 1 |
| Smx |  |  |  |  |  |  | 7 | 6 |
| Tet |  |  |  |  |  |  |  | 6 |
